# From Insight to Action: Applying Community-Based Participatory Research to Improve Population Health Among Black Women

**DOI:** 10.5888/pcd22.240400

**Published:** 2025-05-29

**Authors:** Jennifer Wyatt Bourgeois

**Affiliations:** 1 Center for Justice Research, Barbara Jordan–Mickey Leland School of Public Affairs, Texas Southern University, Houston, Texas

Black individuals have high rates of chronic conditions (eg, heart disease, diabetes, high blood pressure, asthma) and often manage multiple illnesses simultaneously (1). Persistent health challenges among Black women in the United States represent an urgent public health crisis. One-size-fits-all research approaches and traditional interventions have not always taken into consideration the impact that nonmedical determinants of health plays on health and well-being. Community-based participatory research (CBPR) is an approach in which researchers, organizations, and community members can collaborate on all aspects of a research and evaluation effort (2). This essay briefly explores root causes of poor health impacting Black women, highlights the potential of CBPR to address these challenges, and presents strategies to enhance CBPR practices for improving population health among Black women.

## Root Causes of Poor Health Outcomes Among Black Women

Black women constitute 14% of the US female population and 52% of the Black population. Black women experience high death rates from heart disease, cancer, stroke, and diabetes (1). Additionally, despite socioeconomic status, Black women are disproportionately impacted by obesity (3). The prevalence of obesity among Black women increases the likelihood of complications, such as cardiovascular disease, type 2 diabetes, and nonalcoholic fatty liver disease (3). Reported poor health outcomes underscore the need for research and interventions that both explore these issues and engage the affected communities in identifying solutions. Understanding nonmedical determinants of health (transportation, neighborhood and built environment, education) in shaping health is important in recognizing how traditional interventions may not be sufficient (4).

## Why Conventional Approaches to Health Equity Fall Short

Interventions that do not consider the concerns of communities, and those that primarily focus on clinical treatment without taking into consideration previously mentioned nonmedical determinants of health, may fall short in addressing the specific needs of communities. This lack presents opportunities to identify more innovative and comprehensive strategies to help improve the health of Black women. CBPR places emphasis on proactively engaging community members as active partners. This engagement will help ensure that research and interventions are grounded in lived experiences and are tailored to address specific health challenges (5,6).

## The Role of Community-Based Participatory Research

By engaging the community in a transparent and collaborative research process, CBPR is essential in helping to build trust among Black women so that they can take an active role in research focused on their unique health concerns (7–9). A review of 15 CBPR programs, 3 targeting Black women, looked at programs that sought to increase physical activity to address obesity, diabetes, and cardiovascular disease in African American communities. By using various research approaches, these programs achieved modest improvements, such as slight reductions in body mass index and increased weekly physical activity (10). To illustrate the practical application of CBPR and its impact on Black women’s health, the following section describes a 2023 pilot initiative that used this approach to initiate a dialogue to identify strategies to improve population health (11).

## Pilot Study: 2023 Pilot for Black Women’s Health in Texas

The usefulness of CBPR was shown in a pilot involving health and wellness events held in the areas surrounding Houston, Texas. This initiative was designed as an exploratory project, using CBPR strategies (eg, community entry or engagement and health problem identification) to gather initial insights into health challenges faced by Black women. The pilot aimed to collaboratively address health and wellness needs specific to Black women by introducing relevant wellness activities in ways that are tailored and accepted locally. This exploratory pilot initiative sought to identify activities that can enhance mental and physical health outcomes, focusing on reducing stress, increasing physical activity, and fostering community connectedness. These activities were chosen based on research showing their effectiveness in addressing health challenges (12). All activities were led by Black women to foster comfort, validation, and a sense of community (13). Engagement, a key CBPR strategy, was encouraged during the events by wellness practitioners and health professionals holding one-on-one and group conversations with participants, fostering open dialogue about health-related questions and concerns, which provided valuable insights for tailoring future interventions based on community input. This initial stage focused on internal community feedback and engagement; therefore, institutional review board approval was not required. Future phases of the initiative will formally evaluate the impact of these activities on health outcomes, such as stress levels and activity frequency, using specific metrics such as pre-surveys and post-surveys on mental health (eg, stress reduction scales) and physical activity levels, with the goal of scaling up effective strategies.

The aforementioned pilot study serves as an exploratory example to demonstrate the practical application of CBPR principles. Although exploratory and not intended to yield conclusive outcomes, the pilot offered meaningful insights on the importance of working collaboratively to implement tailored wellness activities, including yoga and meditation, as well as strategies rooted in community engagement. Feedback from participants also informed the identification of key barriers to wellness and opportunities for tailored interventions, laying the groundwork for future recommendations focused on improving CBPR practices to improve population health among Black women.

## Improving CBPR Practices

The L.O.T.U.S. (Lead, Optimize, Tailor, Utilize, Sustain) recommendations were developed by this author in 2024 to strengthen CBPR practices in addressing the unique health challenges experienced by Black women. Drawing on general principles of community engagement and lessons from research and evaluation insights, these recommendations aim to refine CBPR applications to better align with the needs of Black women. The L.O.T.U.S. framework provides a structured approach to advancing this objective.

1. **Lead with Black women–centered research.** Engaging Black women to play a key role in identifying solutions to persistent health challenges has proven essential for developing locally driven solutions. For example, the Trust Black Women project used a CBPR framework to prioritize the voices and lived experiences of Black women, broadening the understanding of reproductive health beyond just sexual risk behaviors. This approach provided a comprehensive view of the health concerns and decision-making processes affecting Black women (14).
2. **Optimize resource allocation. **Strategic allocation of resources is essential for the initial success of CBPR efforts focused on Black women. This includes identifying a range of resources for capacity building, fostering partnership synergy, and implementing short-term projects that address the immediate needs of Black women’s health while laying the groundwork for sustainable practices (15).
3. **Tailor interventions to address specific local health challenges.** Recruiting Black women in health research and designing interventions to specifically address health challenges is well illustrated by the Kin Keeper Model (16). This CBPR-based intervention improved cancer prevention and screening by engaging Black women as health educators within their families, increasing cancer awareness and promoting health behaviors through family-centered engagement.
4. **Use continuous community feedback loops.** Community feedback is crucial in CBPR approaches to ensure that research meaningfully addresses the unique experiences of the community, in this case Black women. In prior maternal health research, incorporating a study participant into the analysis phase ensured that the findings were interpreted in a way that resonated with the lived realities of Black women, making research and evaluation efforts more relevant and effective to local settings (17).
5. **Sustain impact with long-term research and evaluation.** Conducting longitudinal studies is essential for ensuring the lasting effectiveness of CBPR interventions aimed at improving Black women’s health. The limited availability of long-term research underscores the importance of evaluations to track health outcomes, address shifting community needs, and refine strategies over time.

## Conclusion

The persistent chronic disease burden among Black women supports the need for immediate and sustained public health action that involves working closely with community members. Researchers and practitioners working closely with members of the community can help create tailored health interventions to meet local needs. For Black women, this means focusing on developing health interventions that take into consideration their lived experiences, build on essential partnerships, improve clinical–community linkages, and encourage family-centered engagement.
